# Retraction: Comparative Impact of Integrated Palliative Care vs. Standard Care on the Quality of Life in Cancer Patients: A Global Systematic Review and Meta-Analysis of Randomized Controlled Trials

**DOI:** 10.1371/journal.pone.0355244

**Published:** 2026-08-04

**Authors:** The PLOS One Editors

Following the publication of this article [1], concerns were raised about the methodology. Specifically:

- The Abstract and the Results section of this article incorrectly report the mean differences (MDs) calculated in this study as standardized mean differences (SMDs) instead.
- The MDs reported in Figs 2-6 appear to have been calculated using different outcome measurement scales, meaning that a change of one unit does not represent the same effect across studies, but the article does not report standardization of effect sizes. In the absence of SMDs for results that compare outcomes measured on different scales, the validity and reliability of the effect sizes reported in [1] are in question.
- The reliability of the data extraction from the studies included in the meta-analysis is in question, specifically for references 16, 18, and 20–21 in [1]. For example, for Reid *et al*. (reference 21 in [1]), the values reported as means in Table 1 and Figs 2-6 in [1] do not correspond to APCA POS scores but instead reflect within-arm percentage improvement.
- In Table 1 and Figs 2-7, the studies “Marie A *et al*. 2015” and “Jennifer S *et al*. 2016” could not be identified in the reference list of [1].

The corresponding author stated that the use of MDs was appropriate and that the term SMD was used mistakenly in the Abstract and Results sections. They also provided screenshots from the source articles to support the accuracy of the extracted data. The corresponding author clarified that “Marie A” refers to Bakitas MA (reference 16 in [1]) and that “Jennifer S” refers to Temel JS (reference 22 in [1]).

An independent Statistical Advisor evaluated the concerns raised and confirmed that the article reports pooled MDs instead of SMDs and that, in light of the heterogenous outcome scales, the MDs should have been standardized before being analyzed. They concluded that the use of MDs instead of SDMs is likely to have had a major impact on the article’s findings. Furthermore, the Statistical Advisor commented that it is unclear how different follow-up times and multiple quality-of-life measures within studies were handled.

In light of the above unresolved concerns that question the reliability and validity of the results in [1], the *PLOS One* Editors retract this article.

AG agreed with the retraction. AE and TAK either did not respond directly or could not be reached.
